# Untested Assumptions and Tenuous Evidence: A Critique of the Dual-Process Account of Moral Judgment

**DOI:** 10.3390/bs16010020

**Published:** 2025-12-22

**Authors:** Philip T. Quinlan, Dale J. Cohen

**Affiliations:** 1 Department of Psychology, The University of York, York YO10 5DD, UK; philip.quinlan@york.ac.uk; 2 Department of Psychology, The University of North Carolina at Wilmington, Wilmington, NC 28403, USA

**Keywords:** computational modeling, dual-process theory, psychological value theory, moral reasoning, trolley problems

## Abstract

The *dual-process theory of moral judgment* asserts that moral judgments come about because of the operation of either of two independent decision processes, often described as a cognitive/rational process and an intuitive/affective process. In some cases, these processes are seen to operate in competition. We trace the development of this account and highlight how the neural and behavioral evidence almost universally relies on the validity of a series of untested statements that, collectively, we call the *dual-process assumptions*. We show how these assumptions produce experimental methods that cannot falsify the dual-process account. We provide an in-depth and critical analysis of the kind of neurophysiological and behavioral evidence that has been used to support the theory and conclude that it is tenuous, equivocal, or both.

## 1. Introduction

A moral dilemma contains a choice that involves matters of “right” and “wrong”. When solving moral dilemmas, researchers often assume that people engage in a unique form of reasoning known as *moral reasoning*. Much of the psychological research surrounding moral reasoning has focused on how participants respond to sacrificial moral dilemmas. Such dilemmas typically ask participants to choose between saving the lives of *n* individuals by sacrificing *n* other individuals. Perhaps the most influential theory of moral reasoning that has emerged from the study of these dilemmas is the *dual-process theory of moral judgment* (henceforth, DPMJ). The DPMJ asserts that moral judgments come about because of the operation of two independent decision processes: a slow, effortful cognitive process (often framed as “consequentialist”, “rational”, “cognitive”, “utilitarian”, or similar) and a fast, effortless emotional process (often framed as “non-consequentialist”, “emotional”, “affective”, “deontological”, or similar; see, e.g., Paxton & Greene, 2010). The DPMJ posits that the cognitive process typically supports consequentialist judgments, while the emotional process typically supports non-consequentialist judgments. The theory is ambitious in attempting to accommodate both brain imaging and behavioral data.

The notion that moral judgment rests on the consideration of two independent systems, which may compete, is central to the DPMJ, and this basic idea has taken a firm grip on the field. Indeed, given the huge impact this theory continues to have on the field, it is important to carefully consider the evidence upon which the theory rests. Here, we provide a focused, critical review of the DPMJ and the data addressing the theory. We show that the DPMJ makes weak predictions and that the supporting evidence rests on a series of untested a priori assumptions (namely, the ‘DPMJ assumptions’) that render the theory unfalsifiable.

## 2. The Foundational Assumptions of the DPMJ Account

In 1973, Newell (one of the founding fathers of cognitivism) questioned the utility of “…the construction of oppositions—usually binary ones.” (Newell, 1973, p. 287) in the development of scientific theories, and the force of his worries is as relevant now as it was then. Over the last two decades or so, work on the psychology of moral reasoning has been fixated on one binary opposition: utilitarianism vs. deontology. Both are considered in detail shortly. However, even the most cursory internet search brings forth a wide range of alternative moralities (for example, see Baber, 2018), such as virtue ethics and cultural relativism, each of which barely receives a mention in the recent psychological literature once the central binary distinction has been introduced. In presenting this, we are arguing not that the distinction between utilitarianism and deontology is irrelevant but merely that theorists have shackled themselves unnecessarily to a rather narrow view of what may operate as the explanans. In simple terms, the utilitarian/deontology opposition is couched in terms of the difference between a consequentialist (utilitarian) view that an action is right or wrong depending on its consequences and a non-consequentialist (deontological) view that certain actions are categorically wrong no matter what the consequences. Typically, the consequentialist view is equated with utilitarianism as espoused by Bentham (1780/1996). The essence of this view is conveyed by the idea that the best action is the one that maximizes utility, whereby utility is defined in terms of happiness or increasing pleasure over pain. The greatest happiness for the greatest number provides a means for gauging right over wrong. The moral worth of any action depends entirely on its consequences and, therefore, the intention behind the action is of no relevance in assessing right or wrong.

The deontological view contrasts starkly with this in stating that certain actions are categorically wrong no matter what the consequences. Such a view is attributed to Immanuel Kant. It is perhaps overly cavalier to attempt to address the underlying issues in shortened form, but here is not the place to worry unduly about the implications of what is being discussed (see Johnson & Cureton, 2018). According to Kant, good actions come about out of respect of moral duty—the application of a “good will”. The categorical imperative states that one must act from duty, following only principles that can be universally applied, regardless of circumstances. One such principle is to never treat a person as a means to an end. In this vein, the intentions behind an action are key, not its consequences. Stated thus, one can identify a logical basis for the utilitarian/deontological binary opposition. This binary opposition has acted as the primary driver of theorizations about moral reasoning in psychology in the recent past.^1^

### 2.1. The Trolley Dilemma

In accepting this binary opposition, the psychology of moral reasoning has advanced primarily by developing empirical tests that pit predictions derived from utilitarianism against those derived from deontology (cf. Newell, 1973). Such tests often examine reasoning about a certain kind of sacrificial moral dilemma known as *trolley dilemmas* (Foot, 1967; Sharp, 1898; Thomson, 1985). A standard form of the trolley dilemma (termed the *switch dilemma*) is as follows:

You are walking alongside a railway track and notice that a train is approaching that appears to be out of control—its brakes have apparently failed. If the train continues on its path, then it will careen into five workmen who are working on the track and inevitably kill them. However, there is a siding that branches well in front of the workmen, and if the train could be diverted down the siding, then the five workmen will be saved. There is a lever close to where you are standing, and if you pull it, you will be able to divert the train down the siding and so save the five workmen. However, there is a single workman on the siding who would be run over and killed by the train.

The question now is “What would you do?”.

In simple terms, reasoning according to utilitarianism leads to the decision to pull the lever and divert the train. This is predicated on accepting the Axiom of monotonicity—more lives have higher value than fewer lives (see D. J. Cohen et al., 2022, p. 2015). By contrast, it has been argued (see Kahane et al., 2015) that reasoning according to deontology leads you to “do nothing”. Such a simple test, therefore, apparently provides an effective way to determine if people’s moral reasoning accords with utilitarianism or deontology. As a consequence, a primary driver of research into moral reasoning has involved presenting trolley dilemmas to participants and categorizing their responses as utilitarian or deontological. Based on this categorization of “do something” vs. “do nothing” responses, researchers have attempted to identify the conditions that influence the likelihood of engaging in utilitarian vs. deontological reasoning. For example, in critiquing the work of Bostyn et al. (2018), Plunkett and Greene (2019) state, “In nearly all research using trolley-type dilemmas, participants evaluate only the proposed utilitarian action (e.g., pushing the man off the footbridge to save five lives) and do not separately assess the deontological alternative (e.g., not pushing). Because participants give only a single judgment, their responses are inherently comparative, accounting for both “horns” of the dilemma.” (p. 1389).^2^

Science is best served when researchers clearly specify their assumptions. When assumptions are not specified, they must be inferred from how experiments are designed, and the ensuing data are analyzed. Critically, when an analysis relies on the categorization of responses as either “Consequentialist” or “Non-consequentialist” (or any other theoretical dichotomy), then it rests on a series of implicit assumptions. If these assumptions are invalid, then the analysis and the conclusions based on them are invalid. Clearly, the consequences can be far-reaching, and given this, it is critical to understand what is required to classify responses as either “Consequentialist” or “Non-consequentialist.”

### 2.2. The DPMJ Assumptions

In analyzing the dual-process account, it is important to distinguish between the *conceptual theory* and the *empirical model* used to test it. We acknowledge that proponents of the DPMJ often describe these processes conceptually with nuance, explicitly rejecting the idea that they function as rigid or deterministic systems. For example, the DPMJ theoretically distinguishes between the cognitive mechanism and the utilitarian output. However, the data cited in support of the theory relies on an a posteriori classification of responses in trolley dilemmas (i.e., categorizing specific choices as inherently ‘Consequentialist’ or ‘Non-consequentialist’). Because this classification is not based on direct observation, it functions as an inference derived from a measurement model that rests on a series of strong a priori assumptions. Furthermore, it mathematically conflates the mechanism and the output, treating the output as a direct proxy for the process. Critically, accepting the resulting data as support for the theory requires accepting these underlying assumptions. Specifically, for the standard interpretation of the DPMJ to be valid, the classification scheme implies the following:

**1. The Exclusivity Assumption:** When presented with a moral dilemma, participants engage in *only* consequentialist and/or non-consequentialist reasoning. This assumption is necessary to exclude all other potential decision-making processes, from simple random choice to a process that is neither strictly consequentialist nor strictly non-consequentialist (e.g., virtue ethics).

**2. The Prescriptive Assumption:** Each reasoning process invariably results in a specific behavioral outcome. For example, consequentialism is assumed to *always* produce the action that saves the larger group (action), while non-consequentialism is assumed to *always* forbid the action of killing (inaction). If this were not the case—for instance, if a consequentialist calculus could sometimes favor the smaller group—the link between choice and reasoning type would be broken.

**3. The Infallibility Assumption:** Both the consequentialist and non-consequentialist reasoning processes are deterministic and executed without error. This is required because if reasoning is fallible, a person intending to make a consequentialist choice could “err” and perform no action, and vice versa. An error-prone process would make it impossible to diagnose the underlying reasoning from the observed response.

These assumptions must all hold true to categorize the choice to act as “Consequentialist/Utilitarian” and the choice to do nothing as “Non-consequentialist/Deontological” (see, e.g., Conway & Gawronski, 2013; Cushman et al., 2010; Greene et al., 2001; Paxton et al., 2012).

To illustrate the fragility of this classification scheme, consider a 50/50 split in responses to the standard form of the trolley dilemma. The traditional DPMJ interpretation would be that 50% of participants engaged in consequentialist/utilitarian reasoning and 50% engaged in non-consequentialist/deontological reasoning. However, this conclusion is only valid if all the above assumptions are true. If we reject the Exclusivity Assumption, the 50/50 split could simply reflect participants responding randomly without engaging in any formal reasoning process. Alternatively, it could reflect several unidentified decision processes that are neither strictly consequentialist nor strictly non-consequentialist (e.g., virtue ethics). It might even reflect a combination of the two (e.g., a set of random responding participants plus a set of participants who engage in virtue ethics). If we accept the Exclusivity Assumption but reject the Infallibility Assumption, the results could be explained by either a single, error-prone consequentialist/utilitarian process or a single, error-prone non-consequentialist/deontological process, each with a 50% error rate. In such a scenario, it would be impossible to distinguish a utilitarian reasoner who made a mistake from a deontological reasoner who executed their choice correctly. Finally, if we accept both the Exclusivity and Infallibility Assumptions but reject the Prescriptive Assumption, both the consequentialist and non-consequentialist processes could lead to the *same* response choice in the standard trolley dilemma. If both processes lead to the same response choice, then response choice cannot distinguish between the two processes. Because DPMJ researchers identify the reasoning process directly from the behavioral response, they are implicitly forced to accept this entire chain of assumptions.

#### 2.2.1. The DPMJ Assumptions Underlie the PD and CNI Models

It might be argued that advanced psychometric approaches, such as process dissociation (PD; Conway & Gawronski, 2013) or the CNI model (Gawronski et al., 2017), resolve these issues by independently estimating the parameters for utilitarian and deontological inclinations. However, a close examination reveals that these models explicitly rely on the DPMJ assumptions to achieve mathematical identifiability.

First, these models rely on the Prescriptive Assumption. To calculate a ‘Utilitarian’ (*U*) or ‘Deontological’ (*D*) parameter, the researcher must determine a priori which specific behavioral output (e.g., ‘Action’ or ‘Inaction’) corresponds to which philosophical construct. Without assuming that ‘Action’ is the prescriptive target of the utilitarian process, the model cannot identify the parameter.

Second, the processing tree logic implies a form of exclusivity at the level of execution. While the model allows an individual to possess both *U* and *D* inclinations (trait independence), the mathematical structure posits that for any given decision, the response is driven by the successful engagement of a single parameter (e.g., if *U* drives the response, then *D* is effectively bypassed in that instance).

Finally, and most critically, these models rely on the Infallibility Assumption. As illustrated by the processing tree in Conway and Gawronski (2013), the model assumes that if the utilitarian process is engaged (probability *U*), it invariably results in the prescriptive utilitarian judgment. The model does not allow for a utilitarian (error-prone) process to be engaged (e.g., attempting to maximize utility but failing to execute the corresponding action). Instead, any deviation from the predicted response is mathematically attributed to the *absence* of the inclination (the 1 − *U* parameter space) rather than to fallibility in the process itself.

The CNI model (Gawronski et al., 2017) attempts to address the limitations of standard PD by adding a parameter for generalized action/inaction (*I*), seemingly decoupling moral inclinations from response biases. However, it retains the Prescriptive and Infallibility Assumptions for its moral parameters: it posits that if sensitivity to consequences (*C*) or norms (*N*) is engaged, it invariably results in the prescriptive judgment without error. Like the PD model, CNI attributes inconsistency strictly to a failure to engage in the moral process rather than to fallibility in the process’s execution. Finally, the Exclusivity Assumption is also retained, but rather than identifying two processes, it identifies three (i.e., *C*, *N*, and *I*).

Consequently, even these sophisticated models cannot disentangle the reasoning process from the response without relying on the very assumptions they aim to test.

#### 2.2.2. The Preclusion of Validity Testing

The a priori acceptance of the DPMJ assumptions manifests itself in several ways and is widespread in the moral psychology literature (Ahlenius & Tännsjö, 2012; Bago & De Neys, 2019; Bostyn et al., 2018; Conway & Gawronski, 2013; Conway et al., 2018; Paxton et al., 2012; Suter & Hertwig, 2011; Tinghög et al., 2016; Trémolière & Bonnefon, 2014). Critically, though, any task that is based on the a priori acceptance of the DPMJ assumptions *cannot also be considered a test of the validity of that account*. What is key here is that the DPMJ assumptions are being used to classify a response as being indicative of a specific kind of reasoning. In critical cases, the classifier is being applied only once the data are in. Moreover, the validity of the classifier itself is never tested. In this regard, the theory, such that it is, provides nothing other than a convenient means for organizing the data obtained from our observations and measurements (paraphrasing Matzkin, 2002, p. 286).

Nevertheless, if the DPMJ assumptions are accepted as valid, one may assert that the relevant experimental tasks are tests of who primarily uses utilitarian vs. deontological reasoning (see, e.g., Koenigs et al., 2007) and/or the conditions under which utilitarian or deontological reasoning is deployed (see, e.g., Greene et al., 2001). However, if the DPMJ assumptions turn out not to be valid, then this argument fails.

The DPMJ assumptions are the foundation of the DPMJ’s empirical claims. In the following sections, we demonstrate how the DPMJ assumptions (1) create circular and equivocal neuroimaging evidence, (2) are directly contradicted by core behavioral findings, and (3) continue to distort modern research practices by obscuring simpler, more parsimonious explanations.

## 3. Critique 1: How the DPMJ Assumptions Create Circular Neuroimaging Evidence

In this section, we show that the foundational MRI evidence for the utilitarian vs. deontological classification is an artifact of the DPMJ assumptions rather than providing support for these assumptions.

### 3.1. Re-Examining the Foundational Evidence: Greene et al. (2001)

In 2001, Greene et al. (2001) published what turned out to be an immensely influential paper on the neuroscience of morality (i.e., the neural underpinnings of moral reasoning). In this paper, Greene et al. (2001) laid the foundations for the DPMJ. Greene et al. (2001) were primarily interested in why participants responded differently in the standard trolley dilemma (which, for expository convenience, we shall refer to as a *switch dilemma*) versus a specific variant of the trolley dilemma often referred to as a *footbridge dilemma*. In the footbridge dilemma, you find yourself on a footbridge over a single track standing next to a very large man, and, as in the standard dilemma, an out-of-control train will kill five workmen on the track if you do nothing. You can push the very large man onto the track, which will kill him but also impede the train’s progress, thus saving the five workmen.

In stark terms, the consequences of action/inaction in the lever and footbridge scenarios are identical; inaction leads to the death of five people, whereas action (either pulling the lever or pushing the man) leads to the death of one person. Nevertheless, people tend to endorse action in the switch dilemma (pull the lever to save more people) more often than in the footbridge dilemma (push the very large man onto the track to save more people). From a careful reading of the paper, Greene et al. (2001) never state the reason why people would save the larger group in the switch dilemma. To them (and presumably the reader), such a justification is self-evident—sacrificial moral dilemmas engage utilitarian reasoning, and this (obviously) provides the rationale for action. The real question that needed explaining, by contrast, was why people *would not* do the same for the footbridge dilemma.

For Greene et al. (2001), the key difference between the switch and footbridge dilemmas lies in the emotional valence of the scenario. Whereas the thought of pushing someone to their death provokes a salient emotional reaction, the corresponding thought of pulling a lever does not. Greene et al. (2001) further hypothesized that taking action in the footbridge dilemma is at odds with the emotional reaction of having to physically push the man, which causes a “Stroop like” conflict.

It is here that Greene et al. (2001) first invoke the Prescriptive Assumption to a priori classify responses. Greene et al. (2001) state, “More generally, we predicted longer reaction times for trials in which the participant’s response is incongruent with the emotional response (e.g., saying “appropriate” to a dilemma such as the footbridge dilemma)” (p. 2106). Here, the authors identify one response as “emotional” (later to be labeled “deontological”), and, through implication, the other response is non-emotional (later to be labeled “utilitarian”).

Across two experiments, Greene et al. (2001) examined, respectively, neural data (fMRI signals, Experiments 1 and 2) and behavioral data (reaction times, i.e., RTs, Experiment 2) for a range of moral and non-moral dilemmas. The authors labeled the switch dilemmas *moral impersonal dilemmas* and the footbridge dilemmas *moral personal dilemmas* (a key distinction in the initial account, see Box 1. Greene & Haidt, 2002). The authors predicted that the “brain areas associated with emotion would be more active” when solving moral personal dilemmas relative to moral impersonal dilemmas (p. 2106).

The neuroimaging data revealed that the medial prefrontal cortex (BA 9 and 10), bilateral angular gyrus (BA 39—revised later to be identified as the bilateral superior temporal sulcus, Greene and Haidt (2002)), and posterior cingulate (BA 31) were more active when participants were responding to the moral personal dilemmas compared to the moral impersonal dilemmas. At the time, these areas were described as being associated with emotion. Further, the data revealed that Regions BA 46 (middle frontal gyrus) and BA 7/40 (bilateral parietal lobe) were more active in impersonal than in personal scenarios. The authors identified these regions as being important in (non-emotional) working memory tasks (J. D. Cohen et al., 1997).

The behavioral data collected in Experiment 2 revealed that participants were markedly slower in making “appropriate” responses to the moral personal (footbridge) dilemmas (push the person off the bridge) than they were in responding to all other dilemma types. The authors interpreted this slowing in a single condition as confirmation of the “Stroop”-like interference.

In sum, the neuroimaging data reveal that scenarios previously identified as emotional engaged areas of the brain associated with emotions. Furthermore, scenarios previously identified as non-emotional engaged areas of the brain associated with working memory. Critically, these results were *scenario-specific* rather than *response-specific*. That is, there was no neuroimaging evidence that the “do something” and “do nothing” responses engaged different brain regions. Furthermore, the behavioral data revealed that participants responded more slowly when responding “appropriate” than when responding “inappropriate” to the moral personal dilemmas. The authors took this as a behavioral manifestation of the (presumably) “Stroop”-like interference that arises when the (presumably) “emotional response” (i.e., “do nothing”) is (presumably) incongruent with that (presumably) favored by (presumably) utilitarian reasoning (i.e., “do something”). Although these results are interesting, they provide almost no evidence for separate, independent decision mechanisms. Indeed, the authors prudently noted that the data identified correlative rather than causal effects: “What has been demonstrated is that there are systematic variations in the engagement of emotion in moral judgment. The systematic nature of these variations is manifest in observed correlations …” (p. 2107). Nevertheless, this story was soon developed into the DPMJ, with neuroanatomical evidence providing its primary support.

### 3.2. The Emergence of the DPMJ: A Priori Classification and Circular Logic

The general framework for a “dual process” account of moral judgment emerged in Greene et al. (2004), where the authors proposed that “both ‘cognitive’ and emotional processes play crucial and sometimes mutually competitive roles” (p. 389). Greene et al. (2004) implicitly accepted the DPMJ assumptions a priori. Once accepted, the authors used linguistic maneuvers to create the impression that the data supports the DPMJ. This reasoning is circular: data that can only be interpreted through the DPMJ assumptions cannot then be used to validate those same assumptions.

Greene et al. (2004) implicitly accept the Prescriptive and Infallibility Assumptions to provide the logical foundation for classifying responses as utilitarian vs. nonutilitarian—which they do throughout the paper (e.g., “To test the hypothesis that …, we compared the neural activity associated with utilitarian judgments (accepting a personal moral violation in favor of a greater good) to nonutilitarian judgments (prohibiting a personal moral violation despite its utilitarian value)” (p. 392)). By labeling the “do something” response as “utilitarian judgments” and “do nothing” responses as “nonutilitarian,” the authors assign causality (e.g., utilitarian reasoning caused the “do something” response) in the absence of evidence. With these assumptions (and illusionary causality) in hand, the authors then classify neural responses as being indicative of a specific kind of reasoning (e.g., “In other words, this hypothesis predicted that the level of activity in regions of DLPFC would correlate positively with *utilitarian moral judgment*” (emphasis added, p. 390)). This is an example where a classifier is being applied only once the data are in, and, critically, the validity of the classifier itself is never tested.

Greene et al. (2004) also apply this tactic to the “do nothing” responses. The authors identify the “nonutilitarian” response as resulting from a “social-emotional” process that prefers the “do nothing” response. For example, the authors state that “some moral judgments, which we call “personal,” are driven largely by social-emotional responses…” (p. 389) and that “… personal moral violations elicit prepotent, negative social-emotional responses that drive people to deem such actions inappropriate,” (p. 390). Again, a classifier is being applied only once the data have been collected, and the validity of the classifier itself is never tested.

Finally, the authors assume that the utilitarian and social–emotional systems are the only two players in the game (the Exclusivity Assumption). By way of example, consider the so-called *crying baby dilemma*. This has been described as a “high conflict” scenario (see Greene et al., 2004) and involves choosing to kill “a crying baby” to save an unspecified but large number of “townspeople”. Greene et al. (2004, p. 393) stated that “… moral dilemmas such as the crying baby dilemma involve a conflict between (1) social-emotional responses that drive people to disapprove of personal moral violations and (2) countervailing “cognitive” processes that drive people to approve of such violations in the relevant contexts”. By committing each of the two response options to separate, respective processes, the authors exclude the possibility of other processes producing the responses. This final assumption (i.e., Exclusivity) is required for the response/reasoning classifications to be made with confidence. With this, the DPMJ is complete.

In the associated research, the neuroanatomical data are used to provide support for the primary prediction “…that different patterns of neural activity [those associated with social-emotional vs. utilitarian reasoning] in response to the same class of moral dilemma [personal moral dilemmas] are correlated with differences in moral decision-making behavior [the ‘do something’ vs. ‘do nothing’ response]” (parentheses added, Greene et al., 2004, p. 390). That is, the authors are looking for two different neural profiles: one that is active when people judge saving the larger group as appropriate in personal moral dilemmas, and another one that is active when people judge saving the larger group as inappropriate in personal moral dilemmas. Such profiles, if previously correlated with similar cognitive tasks (e.g., abstract reasoning and/or cognitive control vs. social and emotional), are then taken as evidence supporting their theory.

Initially, the data appeared to support the authors’ hypotheses. An increase in the activation of the DLPFC was found when people judged saving the larger group as appropriate in personal moral dilemmas. The authors claimed that the DLPFC reflects “engagement of abstract reasoning processes and cognitive control” (p. 390). Greene et al. (2004) placed particular emphasis on what they labeled the “anterior dorsolateral prefrontal cortex (BA 10/46)”, which they considered to play a key role while one is engaged in reasoning about difficult as opposed to easy personal moral judgments.^3^ The authors do not report findings that identify an increase in the areas of the brain that are associated with social–emotional reasoning when people judged saving the larger group as inappropriate in personal moral dilemmas (they rely on Greene et al., 2001, for that evidence).

To test their claim that conflict arises because the different processes deliver different decisions about the appropriate course of action in the personal moral dilemma, Greene et al. (2004) assessed the activity of the anterior DLPFC and the anterior cingulate cortex (ACC). The anterior DLPFC has been previously associated with abstract reasoning, and the ACC has been associated with conflict (see, for instance, Botvinick et al., 1999), with the possibility that it is responsible for conflict monitoring (Botvinick et al., 2001). Greene et al. (2004) suggest that increased activation of both regions is required to overcome the influence of the (presumed) social–emotional process and choose the (presumed) preferred (presumed) utilitarian response in difficult personal moral dilemmas. Evidence in support of the predictions is described in the paper.

In the eventual dual-process theory (see Figure 2 in Greene, 2009b), two neurological routes are posited. The affective route comprises the amygdala/superior temporal sulcus/TPJ, which brings about an initial intuitive appraisal, followed by a deontological/nonutilitarian intuitive emotional response in the medial PFC, BA 9/10 posterior cingulate cortex/posterior STS/TPJ. By contrast, the cognitive route comprises the DLPFC demarked as BA 46—this is where utilitarian moral reasoning takes place—and the DLPFC demarked as BA 10 (otherwise referred to as the anterior DLPC). The account acknowledges that these two routes may result in competition in cases where the different routes produce different decisions. Such cases are signaled by activity in the ACC—the conflict monitor. The neuroimaging and behavioral data reported in Greene et al. (2001) and Greene et al. (2004) became the evidentiary foundation that supports the DPMJ. Given this, it is important to examine how the neuro-psychological account has held up over time.

### 3.3. Reassessing the Neuroimaging Data: Reverse Inference and Post Hoc Maneuvering

Here, we review the evidence that the neuro-psychological account supporting the DPMJ has succumbed to the fundamental problems of “reverse inference” (see, for instance, Klein, 2011). Specifically, the approach adopted by Greene and colleagues has been to “identify brain regions which are active during a particular kind of moral task and then treat these activations as evidence for a particular cognitive theory” (p. 146). Klein emphasizes that reverse inference is problematic when the brain regions involved are pluripotent—that is, when they are implicated in a variety of different processes. Greene and Haidt (2002) acknowledged this when they stated, “many brain areas make important contributions to moral judgment although none is devoted specifically to it.” (p. 517). When the activation of the region is not a reliable indicator of a particular function, reverse inference is compromised. More particularly, according to Klein (2011), if the brain regions are not associated with a specific function concerning emotion or cognition, then the neuroimaging evidence is “an extremely weak test of the dual-track hypothesis” (p. 146).

#### 3.3.1. The Default Mode Network

Greene and Haidt (2002) aligned the regions associated with emotion (i.e., the medial prefrontal cortex, posterior cingulate/precuneus, and superior temporal sulcus/temporoparietal junction) with what they described as the network associated with the resting state of the brain (Gusnard & Raichle, 2001). Raine and Yang (2006) marshalled a variety of evidence that shows that the network is consistently activated in tasks that bear on moral information processing. The details are scant, but the argument made in Greene and Haidt (2002) appears to be that the activation of this network may be taken as a marker of engagement in introspection and in drifting off task and that people may engage in personal ruminations that bear some relation to the kinds of “high-level social-emotional processing involved in moral judgment” (p. 522).

More recently, however, this particular set of ROIs is discussed in terms of the *default mode network* (Raichle, 2015). Although such a network has typically been discussed as featuring in off-task activity (Gusnard & Raichle, 2001) and being most active during periods of “passive control conditions than active target tasks” (Buckner et al., 2008, p. 3), recent evidence suggests a role for this network during periods of working memory maintenance (Sormaz et al., 2018). The evidence Sormaz et al. (2018) present revealed that activity in the network relates not to the “emotional tone of the experience” (p. 9321) but rather to the level of detail being borne in mind. Clearly, what the functional role of this network is continues to be a topic of intense interest. Questions remain about the degree to which activity in the network signals domain-general operations or particular operations concerning moral information processing (for a much more detailed and critical consideration of these issues, see Klein, 2011).

#### 3.3.2. The DLPFC and VMPFC

More controversy has surrounded the function(s) of the prefrontal cortex. First, it is worth noting the distinction Greene (2009b) drew between utilitarian moral reasoning as enacted in BA 46 and utilitarian cognitive control as enacted by BA 10. Evidence for this distinction is cited but is contained in two sources that remain unpublished. Up until that point (i.e., 2009), the evidence from Greene et al. (2001) and Greene et al. (2004) was that BA 46 was more active when confronted by impersonal dilemmas compared to personal dilemmas—nothing is written about the possible role that BA 46 plays in utilitarian moral reasoning. Indeed, as Moll and de Oliveira-Souza (2007b) pointed out, BA 44/46 was “not found to specifically support utilitarian choices in high conflicting moral judgments” (p. 323) by Greene et al. (2004).

Perhaps more concerning, though, is the evidence from brain-damaged individuals reported by Koenigs et al. (2007). Six patients with damage to the VMPFC were tested on their judgments about moral dilemmas adapted from Greene et al. (2004). Critically, the damage was with respect to what Greene described as the anterior DLPC. A key finding was that the patients endorsed more utilitarian decisions in high-conflict personal dilemmas (push the large man in the footbridge dilemma) than did matched control participants.

Moll and de Oliveira-Souza (2007a) interpreted this finding as undermining the dual-process theory. If the anterior DLPC is responsible for decision-making favoring utilitarian outcomes, then the patient finding is the opposite of what might have been predicted. In reply, Greene (2007) argued that, on the contrary, what was critical was damage to the medial regions of the prefrontal cortex. Damage here compromised the brain’s emotional reaction to the high-conflict personal dilemmas, and without this, there is no conflict. Hence, the lack of activity in the anterior DLPFC is merely a reflection of the lack of the need to suppress a countervailing emotional response. As a consequence, the focus shifts away from BA 10 to BA 46, which Greene (2007) claimed was preserved and capable of utilitarian reasoning. Moll and de Oliveira-Souza (2007b) remained unconvinced by this maneuver and noted the lack of evidence for such a claim given the results reported by Greene et al. (2001).

As with attempting to understand the functional role of the default network, problems abound in attempting to understand the functional role of the DLPFC. It appears that this region has been implicated in working memory (Wager & Smith, 2003) and decision-making in general (Coutlee & Huettel, 2012). In addition, alternative claims have been made about how it may be implicated in moral reasoning (Rosenbloom et al., 2012). What is clear, however, is that the picture is now more complex than before, and issues remain in attempting to align different patterns of DLPFC activity with the particularities of moral reasoning as opposed to reasoning in general (see Klein, 2011).

More recently, the dual-process theory has itself been extended to accommodate a role for the ventromedial prefrontal cortex (vmPFC—see Greene & Young, 2020; Shenhav & Greene, 2010, 2014). This region was identified as being key to cognitive controlling mechanisms responsible for modulating intuitive appraisal, the central claim being that “the vmPFC is preferentially engaged when emotional responses and explicit rule-based must be integrated to form an “all things considered” judgment.” (Shenhav & Greene, 2014, p. 474). This is a region not previously identified as being relevant, and it is unclear how it should be incorporated into the information-processing account.

#### 3.3.3. The ACC

Another example of how untested assumptions drive biased interpretations is evidenced in the interpretation of ACC brain data. Although, in the original account of the moral brain (Greene & Haidt, 2002), no mention is made of the ACC, this region is picked out as worthy of discussion by Greene et al. (2004). In comparing brain activity in difficult vs. easy personal moral judgments, the fMRI analysis revealed increased activity in region BA 32 (the ACC). Greene et al. (2004) argued that difficult personal moral dilemmas provoke a conflict between (presumably) “social-emotional responses” that indicate a (presumed) disapproval of (presumably) personal moral violations and (presumably) “countervailing ‘cognitive’ processes towards approving such violations in the relevant contexts” (p. 393). In this respect, there is conflict, and, according to Botvinick et al. (2001), the ACC acts as a conflict monitoring device that adjusts response biases according to an appraisal of the conflict.

Greene et al. (2004) admit that the conflict monitoring hypothesis is not without controversy, and it is important to note that the interpretation of the behavioral data used to motivate claims about conflict monitoring has been shown to be no longer creditable (see Davelaar & Stevens, 2009; Schmidt & De Houwer, 2011). Greene et al. (2004) went on to discuss alternative accounts of the functional role of the ACC, for example, that the ACC is an error detector (Carter et al., 1998). Greene et al. (2004) argue that the account is not helpful because responses to the dilemmas cannot be described as errors. We find this argument unconvincing. As D. J. Cohen and Ahn (2016) pointed out, error-free processes are unlikely to manifest in a biological system. If one rejects the Infallibility Assumption, then utilitarian reasoning (for example) would be error-prone. Error-prone utilitarian reasoning could result from a variety of processes, including stochasticity, uncertainty in determining what is best for all, etc. An error-prone utilitarian system will choose the “do something” option when it is “correct” (assuming, as researchers mostly do, that this is the preferred option) and the “do nothing” option when it is “incorrect”. Within such a system, error checking would be advantageous. Such a system, however, would be problematic for researchers who accept the DPMJ assumptions because this would prohibit assigning the reasoning process based on the chosen option (see D. J. Cohen & Ahn, 2016). Regardless, error checking is a valid and, indeed, a likely process for an error-prone system to engage in. Thus, the activation of the ACC would more likely be interpreted as error checking rather than conflict monitoring if one accepted an error-prone process (i.e., rejected the Infallibility Assumption).

#### 3.3.4. Post Hoc Maneuvering

By assuming key aspects of the theory as valid a priori and admitting that the manifestation of the theory in the brain is “complex”, researchers can generate seemingly consistent post hoc interpretations of virtually any pattern of brain activation. For example, Greene et al. (2004) reported data that were taken to reveal that brain areas associated with emotion predict “utilitarian” responding (BA 23/31, p. 397)—a result that appears to contradict the dual-process theory. Nevertheless, the authors discounted this this evidence by stating, “This finding does not necessarily undermine our suggestion that ‘cognitive’ processing tends to favor utilitarian judgment in response to the dilemmas employed here. It does, however, challenge the overly simple view that utilitarian judgments are wholly allied with “cognition” while nonutilitarian judgments are wholly allied with ‘emotion.” (p. 397).

A similar scenario occurred in the later paper by Shenhav and Greene (2010), where they reported that the lateral orbital frontal cortex (OFC/vmPFC) exhibited a “significant parametric increase in BOLD activity with increased tendency towards utilitarian judgment at the individual level.” (p. 670). This was an unpredicted result, particularly as, previously, the key regions in this regard were taken to be the DLPFC demarked as BA 46 and BA 10. Neither of these regions was picked out, but the OFC was. Shenhav and Greene (2010) do not shy away from discussing this rather awkward pattern of findings (pp. 673–674) and provide several post hoc explanations for the result (e.g., “implicit modulation of affective representations”, “robust negative representations of the deaths”, etc.). These are clear examples of how unconstrained neuro-theorizing simply changes to accommodate whichever brain region is found to be activated under relevant experimental conditions.

In sum, the neuroimaging evidence cited in support of the DPMJ suffers from fundamental flaws rooted in the theory’s untested assumptions. The reliance on reverse inference for pluripotent brain regions (like the DMN and DLPFC), coupled with consistent post hoc maneuvering to explain away contradictory findings (from VMPFC patient data to unpredicted OFC activations), violates a key principle of scientific theories: a scientific theory must be falsifiable. As Klein (2011) argued, without functionally specific neural correlates, the neuroimaging evidence remains ‘an extremely weak test of the dual-track hypothesis’. Less charitably, the foundational neuro-psychological support for the DPMJ appears invalid.

## 4. Critique 2: Behavioral Predictions and Relevant Evidence

One of the signatures of the strength of any scientific theory is the degree to which relevant evidence supports its predictions. Although the initial indications were that the neuroanatomical predictions of the DPMJ were supported (see, e.g., Greene et al., 2001), with the passage of time, the situation has changed. In the following, we examine key behavioral predictions of the theory and how these have fared with respect to the accumulation of relevant evidence.

### 4.1. Ambiguous Theory and Predictions

Recall that the DPMJ proposes that a slow, rational process that favors the utility-maximizing outcome competes against a fast, social/emotional process that favors the characteristically deontological outcome. Because the DPMJ is described in broad terms rather than as a computational cognitive model, it is impossible to make a priori point predictions for RT and/or choice. For example, the theory provides no information about the mechanistic process that describes the “competition” between the two reasoning systems (e.g., is it a horse-race model, a sequential model, etc.?). Furthermore, there is no objective mechanistic process for a priori predicting when the influence of the social/emotional process manifests. There is the general statement that “high conflict” dilemmas tend to invoke such a state of affairs, together with the contingent claim that these bring about protracted RTs. But the details of what constitutes a “high conflict” dilemma and why slow responding comes about remain elusive. Given the lack of precision in its description, much of the theory is left to be freely interpreted by individual researchers. Such subjectivity leads to flexibility in interpreting data, and such flexibility compromises the falsifiability of the theory. Nevertheless, researchers have reached a consensus on two broad, directional predictions to test the theory.

**Prediction 1**: When the social/emotional process is activated, the utility-maximizing responses should be slower than the characteristically deontological responses. This is because the social/emotional process favors the characteristically deontological outcomes (whereas the rational process does not), and it is faster than the rational process.

**Prediction 2**: When the social/emotional process is activated, increased cognitive load should influence the speed and number of utility-maximizing responses. Such changes in cognitive load ought not to affect the characteristically deontological responses. This pattern is to be expected because the utility-maximizing responses result from the rational process, which requires working memory, and the characteristically deontological responses result from the social/emotional process, which does not.

Critically, even if both predictions were borne by the data, such evidence remains equivocal with respect to the DPMJ. This is because the predictions are both weak and subjective. The predictions are weak because they are directional rather than point predictions. Whereas point predictions are supported by a strictly limited number of patterns of data, directional predictions can be supported by many patterns of data. As such, directional predictions are much harder to falsify than point predictions (see Meehl, 1967). This failing is amplified when one uses null hypothesis statistical testing (NHST) because of the interpretive ambiguity and sensitivity to power of NHST (Meehl, 1967; Szucs & Ioannidis, 2017).

The predictions are subjective because they rely on appeals to the ‘activation of the social/emotional process’. Because there is no objective mechanistic process for a priori predicting the activation of the social/emotional process, individual researchers are free to decide which circumstance gives license to claims about the activation of it. Worse still, if the predicted result is not obtained, the individual researcher can then claim that their experiment failed to activate the social/emotional process. Such flexibility in interpretation eliminates the falsifiability of these predictions (Meehl, 1967).

It is also striking to consider the number of critical issues about processing that the DPMJ remains silent on. Consider the following cases.

1. When the social/emotional process is not activated, what determines the speed and choice of responses? The DPMJ informs us that the rational decision process prefers the utility-maximizing option. The DPMJ does not a priori predict when, why, or how often a characteristically deontological option will be chosen if/when the social/emotional process is not activated. This issue becomes contentious when one considers that many researchers accept the DPMJ, which assumes that the rational process (and the social/emotional process) is error-free. However, if the rational process is error-free, then one would predict that when the social/emotional process is not activated, 100% of the responses would be utility-maximizing. This prediction is clearly not supported. As such, an inconsistency arises because of a lack of mechanistic clarity.

2. What determines the utility-maximizing option? Most researchers have used the quantity of lives saved as the identifier of the utility-maximizing response (see, e.g., Greene et al., 2008). However, others have argued that this is not the best measure (see D. J. Cohen & Ahn, 2016; D. J. Cohen et al., 2022). Without an objective specification of utility maximization, the predictions of the DPMJ rest on the subjective inclinations of the researchers. Such subjectivity undermines the falsifiability of the theory.

3. What determines the activation of the social/emotional process? Without an objective mechanistic account of the activation of the social/emotional process, the predictions of the DPMJ that rely on this process rest on the subjective inclinations of the researchers. Again, such subjectivity undermines the falsifiability of the theory.

4. What determines the speed and choice of response for the social/emotional process? Does the social/emotional process always produce a “do nothing” response, or does it sometimes produce a “do something” response? If so, when and why? Is this process error-free, or is it error-prone? Specifying the mechanistic process in quantitative terms is, again, critical for making key predictions. Without such specifications, the predictions of the DPMJ that rely on this process rest on the subjective inclinations of the researchers. Yet again, such subjectivity undermines the falsifiability of the theory.

5. What is the nature of the competition between the rational and social/emotional process? Even if one fully specifies the nature of the rational and social/emotional processes in isolation, the response produced will depend as much on the nature of the competition process as it does on the nature of the individual processes that are competing. As such, without specifying the competition process, a priori predictions cannot be made.

In sum, although the DPMJ has emerged as the preeminent account of moral reasoning in the psychological literature, there remain a substantial number of critical issues that it simply fails to address. Below, we review how well the data support Predictions 1 and 2.

### 4.2. Prediction 1: Effects on Response Speed and Type of Response

Greene et al. (2001) discussed Prediction 1 in the following way. The authors assumed that the personal dilemmas conjure up an emotional reaction against taking an active role in the killing (thus activating the social/emotional processes) and that responses to endorse such action should be particularly slow “as a result of this emotional interference” (p. 2106). Here, Greene et al. (2001) are stating, without a quantitative description, that the two processing channels (reasoning and social/emotional) are *non-independent*, and they are implying that the system employs an exhaustive stopping rule. Critically, only very particular competition models will produce interference. For example, a horse-race model between two independent channels would not in itself produce interference. For such an interference to arise between two channels of differing speeds (one fast and one slow), one must assume that the system employs an “exhaustive” stopping rule, whereby the system will not respond until both non-independent channels have produced an outcome. Otherwise, the faster channel will (nearly) always produce the outcome first, thus never producing the proposed interference (see Townsend et al., 2007, for a thorough discussion of these issues).

If Greene et al. (2001) adopt an exhaustive stopping rule, as implied, then the system will not produce very fast “do nothing” responses because the system must wait for the slower channel to complete processing before producing a response. This, of course, conflicts with the prediction that the “do nothing” responses are quick because they result from the social/emotional process. Furthermore, unless otherwise specified (quantitatively), interference should be symmetrical across channels. Therefore, *all* responses will suffer from interference in conditions where the two channels produce different outcomes, regardless of which response is produced. Again, such a model predicts that slow “do nothing” responses result from interference, just like slow “do something” responses. There are, of course, a variety of quantitative models that Greene et al. (2001) could have specified, each of which produces a variety of predictions regarding how the two channels interact. These predictions are often complex and unintuitive. Unfortunately, Greene et al. (2001) did not specify a model in quantitative terms, and when examined closely, it is difficult to identify the source of the predictions. It may be that Greene et al. (2001) derived their predictions from intuition rather than quantitative modeling. Such intuitions are out of step with state-of-the-art modeling in this regard.

These substantive issues regarding the nature of the competition process have never been fully addressed. Nevertheless, as noted above, Greene et al. (2001) found that RTs to ‘appropriate’ responses (in which the “do something” choice is endorsed) in personal dilemmas were particularly slow. Against this, though, McGuire et al. (2009) examined performance as a function of the individual dilemmas, and an items analysis revealed that the effects were being driven by the inclusion of a small set of dilemmas. More specifically, a small number of the dilemmas gave rise to “inappropriate” responses in all but a very small number of participants (<5%). Once these so-called poorly endorsed cases had been removed from the data, a new analysis revealed a pattern of responding that no longer fit with the predicted pattern. The data merely revealed that, overall, participants were slower in judging personal moral dilemmas relative to impersonal moral dilemmas (an effect also replicated by D. J. Cohen & Ahn, 2016). McGuire et al. (2009) concluded that the “underlying mechanism responsible for the dilemma type by response interaction … is the opposite of what Greene et al. suggest” (p. 579).

A subtly different approach to these issues has been taken by Bago and De Neys (2019), for they were less concerned with competition processes and more with the idea that the slower rational system operates to correct (i.e., override) the fast intuitive system. They developed a two-response paradigm in which participants were presented with reasoning problems and were instructed to respond rapidly with the first response that came to mind, and, once having done this, they were to respond again, having undertaken a more careful appraisal. In accepting some of the tenets of the DPMJ, responses were classified as being either ‘utilitarian’ (U) or ‘deontological’ (D), and the main interest was the prevalence of the different responses and the degree to which the first and second responses were in agreement.

Some of the data were in line with the idea that a fast deontological response could be overridden by a slower utilitarian response; that is, approximately 11% of the pairwise responses were classified as D then U (i.e., DU). However, in 10% of occasions, the pattern was DD, showing that not all initial D responses were overridden. Of perhaps more interest, though, was the very high number of cases (~74%) where an initial U response was then followed by a second U response (UU cases). Indeed, when the incidence of UU patterns was considered a function of all second U responses (i.e., UU + DU), then the prevalence of UU responding was 87.5%. This was taken as evidence that ‘intuitive’ U responses are emitted very rapidly and not just as a consequence of ‘correction’ by a slower rational system. In sum, even if the idea is that a slower rational system overrides a faster intuitive system rather than that the two systems compete, the relevant evidence still fails to support such a dual-process interpretation: the speed of response is not a useful classifier of the kind of response.

Two further studies are worthy of note, and neither one replicates the patterns of responding found in the data by Greene et al. (2001); hence, neither provides support for Prediction 1. In a complex study regarding, among other things, working memory capacity and executive control in moral judgment, Moore et al. (2008) reported that although “appropriate” responses to personal dilemmas were slower than “inappropriate” responses to personal dilemmas, responses to impersonal dilemmas were slower than responses to personal dilemmas. Moreover, the slowest responses were “inappropriate” responses to impersonal dilemmas. This general pattern was replicated by Moore et al. (2011) in one study, but a slightly different pattern emerged in another (i.e., there was no slowing in “appropriate” responses to personal dilemmas). In sum, the data show that participants were slower to respond to impersonal dilemmas than to personal dilemmas and that the “inappropriate” responses (the “do nothing” option) to the impersonal dilemmas were the slowest responses. There is no obvious explanation in dual-process theory for these findings. Indeed, Moore et al. (2008) conclude that their data “do not support the idea that that executive control must override prepotent emotional processes if people are to endorse provocative resolutions to moral dilemmas.” (p. 556).

In following up on some of these issues, Greene (2009a) acknowledged and, in the main, accepted the points made by McGuire et al. (2009). In reply, he shifted the emphasis away from the distinction between personal and impersonal dilemmas to the distinction between utilitarian and deontological judgments. Greene (2009a) appealed to the fact that later work had focused on performance in high-conflict personal dilemmas (Greene et al., 2004). This, of course, is an example of how the subjectivity inherent in DPMJ predictions compromises falsifiability. Despite Greene’s (2009a) maneuver, further issues were exposed by Kahane and Shackel (2008). They examined the set of moral dilemmas used by Koenigs et al. (2007) that were themselves adopted and adjusted from the original Greene et al. (2001, 2004) studies. When these materials were given to five professional philosophers, only 45% of the impersonal dilemmas and 48% of the personal dilemmas were found to be ones that involved a choice between utilitarian and deontological options. In a later paper, Kahane and Shackel (2010) went so far as to conclude that the set of dilemmas “is highly unlikely to capture any natural distinction in moral psychology, let alone to shed much light on the dispute between utilitarians and their opponents” (p. 570).

A more particular examination of Prediction 1 has been carried out by Gürçay and Baron (2017; see also Baron & Gürçay, 2017). In their experimental tasks, various different kinds of motor responses were measured (e.g., keypresses, mouse movements) and various kinds of personal and impersonal dilemmas were tested. The primary data of interest were the incidence of utilitarian responding—the “do something” choice—and response time. The data Gürçay and Baron (2017) reported was best explained by an alternative to the DPMJ. The alternative account put forward by Gürçay and Baron (2017) was “that both utilitarian and deontological responses to moral dilemmas are typically both available and in conflict with each other.” (p. 54) and that any response reflects a comparison process that weighs these competing considerations. In this account, any claims about differences in the time course associated with the respective responses were dispelled. That is, information pertaining to both responses accrues in parallel, and there is no difference in time course between the two systems. Moreover, in a meta-analysis reported by Baron and Gürçay (2017), the basic dual-process theory predictions were not supported; that is, there was no convincing evidence for a fast deontological process being sometimes overridden by a slower rational utility-maximizing process.

### 4.3. Prediction 2: Effects Related to Increasing Task Demands

In the rejoinder to McGuire et al. (2009), Greene (2009a) made reference to a different behavioral experiment in which RTs were taken when participants responded to moral judgments under various levels of cognitive load (Greene et al., 2008). Prediction 2 was discussed in the following context. The hypothesis was that, with the high-conflict personal dilemmas (of the footbridge kind), endorsing the “do something” option (pushing the man off the bridge) would be particularly impeded by the introduction of cognitive load. Such judgments depend on cognitive control processes that themselves would be compromised by a concurrent load. To test this idea, the following new paradigm was developed.

In each trial, the participant was presented with a written dilemma that was revealed character by character, left to right on the screen. Beneath this, a stream of single digits scrolled across the screen. In the *no load* condition, participants made a Yes/No (kill/save) RT keypress response upon reading the dilemma. In the *load* condition, participants made the Yes/No response but also monitored the stream of digits and made a different keypress response upon detecting the presence of a “5” in the stream. The prediction was that the load manipulation would result in “increased RT and/or decreased frequency for utilitarian moral judgment” (i.e., endorsing the “do something” option, p. 1147). The RT data were clear in showing exactly this pattern. In addition, there was no effect of cognitive load when participants endorsed the “do nothing” option. The data failed to show the predicted pattern when the number of different judgment choices (i.e., “do something” vs. “do nothing”) was analyzed. That is, the predicted effects were revealed in the measures of response speed but not in the prevalence of the different response types. Overall, there is, therefore, only partial support for the predictions of the DPMJ.

As with the original neuroimaging data, many years have passed since the cognitive load paper was published, and it is interesting to note how the findings have fared in light of more recent work. The evidence is mixed. The prediction, set out by Greene et al. (2008), that the endorsement of the “do something” option ought to diminish under conditions of high cognitive load was addressed in the earlier study by Trémolière and Bonnefon (2014). What is particularly distinctive about this study is that Trémolière and Bonnefon varied the kill/save ratio across two critical dilemmas. In one version, the kill/save ratio was (the standard) 1:5, and in another, it was 1:500. The thinking was that, with such extreme kill/save ratios, participants will endorse the “do something” option both quickly and efficiently (hence, these were termed efficient kill–save ratios). As a consequence, any effects of cognitive load should be much smaller in these cases than in the standard 1:5 case. In this experiment, cognitive load was defined in terms of visual short-term memory load. At the beginning of each trial, an array of dots was presented. Next, a dilemma was presented that demanded a response. Finally, the participant had to reproduce the exact position and number of dots as presented in the original dot matrix. Cognitive load was varied as a function of the number of dots presented in the original matrix.

When comparing across the two load conditions for the 1:5 cases, participants endorsed the “do something” option less often in the high load condition than in the low load condition. There was no corresponding load effect in the data for the 1:500 cases. Cast in this way, the data accord with the idea that the so-called efficient kill/save ratios promoted endorsements of the “do something” option and were unaffected by load (or time pressure, as shown in a later experiment).

Gawronski and Beer (2017), however, take a contrary position in examining the work of Trémolière and Bonnefon (2014). The authors argue that the data reveal no effect of the kill/save ratio under the low load conditions. The effect of the kill/save ratio is only obtained under conditions of high load. Similarly, the effect of the kill–save ratio was only obtained under conditions of time pressure to respond and not when the time to respond was unlimited. On these grounds, Gawronski and Beer (2017) claim that the data actually reveal that increasing load and applying time pressure resulted in an increase in “do something” endorsements, in stark contrast to the claim that “do something” endorsements are the result of effortful cognitive processing.

More recently, Tinghög et al. (2016) failed to find any supporting evidence for Prediction 2 in their own study. By previous standards, theirs was a very high-powered study—involving over 1400 participants. The authors focused on testing moral judgments while manipulating cognitive load or time pressure to respond. Earlier work had provided some promising indications that pressing participants to respond quickly resulted in a decrease in “do something” endorsements in high-conflict personal dilemmas (Suter & Hertwig, 2011). Such a pattern is consistent with the idea that, if time is of the essence, then the immediacy of the social/emotional process will prevail because the rational process, being slower acting, will fail to run its course (see also Cummins & Cummins, 2012). Without going into details, neither cognitive load nor time pressure had any effect on the incidence of “do something” endorsements in the study by Tinghög et al. (2016), and given the size of the samples tested, the failures to find any effects cannot be explained away because of low statistical power.

In sum, the DPMJ is described in broad terms rather than as a computational cognitive model. As such, researchers have only derived and tested weak directional predictions that often rely on the subjective interpretation of when the social/emotional process should be activated and/or intuitive, unstated assumptions about the competition process. From our consideration of the relevant literature, the evidence pertaining to the few predictions that have been derived from the DPMJ is contradictory, tenuous, and/or equivocal.

Given the weak neuroanatomical and behavioral evidence, one wonders why the DPMJ remains so prominent in the literature. We present one possible answer to this question below.

## 5. Critique 3: How the DPMJ Assumptions Distort Modern Research

We suspect that the DPMJ endures because it has become an accepted practice to classify the “do something” and “do nothing” responses as utilitarian and deontological, respectively. Such classification accepts the DPMJ assumptions and, by proxy, accepts the DPMJ. Thus, the habitual classification of responses as utilitarian and deontological produces a habitual acceptance of the DPMJ. In making this point, we focus on a recent paper by Bostyn et al. (2018) that has generated considerable interest (Bialek et al., 2019; Colman et al., 2019; Plunkett & Greene, 2019).

The primary issues that Bostyn et al. (2018) address are (i) whether people respond similarly in hypothetical vs. real-life scenarios and (ii) to what degree performance on the surveys predicts actual responses to the dilemmas. In the real-life condition, participants were tested individually in a laboratory in which an electroshock machine was hooked up to two cages: one contained five mice, and the other contained a single mouse. Participants were told that the five mice would receive a non-lethal shock in 20 s if they did nothing but that they could press a button and divert the shock to the single mouse. The measure of interest was how many participants pressed the button—thus producing the “do something” response—before the delay expired. Participants in the hypothetical group were confronted with the same dilemma, but, in this case, the scenario was presented in the form of a textual description. Prior to this stage of testing, the same participants completed a series of surveys, some of which were targeted at measuring “an increased likelihood of consequentialist decision-making on hypothetical dilemmas” (p. 1088). The results showed “that participants were more than twice as likely to make a deontological decision (vs. a consequentialist one) when faced with the hypothetical dilemma (34% of decisions) than they were when faced with the real-life version (16% of decisions)” (p. 1089). Furthermore, the decisions in the real-life scenario were not correlated with the survey measures.

Bostyn et al. (2018) interpret their data consistently with an a priori acceptance of the DPMJ (e.g., “To us, this indicates that when participants made an actual consequentialist decision, this was not driven by anti-social tendencies or by a lack of empathy but by a genuine concern for the greater good”, p. 1091) and question tangential factors, such as the relevance of the surveys and their ability to predict real-life situations (e.g., “The current study therefore suggests that the theoretical importance of these associations might be overrated and that some of these associative patterns are potentially a side effect of the hypothetical nature of traditional research but are not related to *consequentialist moral reasoning per se*”, emphasis added, p. 1090). The authors refer to the participants’ responses repeatedly as “consequentialist” and “deontological” and to the systems that produce the responses as “consequentialist reasoning” and “deontological reasoning”. Even when the authors question a strict interpretation of the DPMJ, they do not question whether people use consequentialist vs. deontological reasoning but rather merely question just *when* consequentialist reasoning is more likely to be employed.

Critically, the Bostyn et al. (2018) experiment illustrates how the DPMJ assumptions drive the questions being studied and the interpretation of the data. By accepting the DPMJ assumptions, the association between the responses and the assumed reasoning processes are taken to be true and are, therefore, not tested. Readers are left with the impression that the DPMJ is, in fact, true and not up for discussion. Researchers, by contrast, examine questions regarding *when* each reasoning process is employed or *who* uses each reasoning process. Indeed, in reply, Plunkett and Greene (2019) took issue with researchers asking the “who” questions, arguing instead that the “when” question was where the real science should be directed.

Here, we assert that the DPMJ should not be reflexively accepted. Indeed, if one rejects the DPMJ, then the conclusions concerning *when* the reasoning processes were switched and *who* switched reasoning processes in the real-life vs. hypothetical conditions fade away, and an entirely different picture emerges. To illustrate, rather than refer to the response options as consequentialist and deontological, we will refer to them as Option A vs. Option B. For example, Bostyn et al. (2018) have provided evidence that, when presented with the same scenario, people are more likely to choose Option A in real-life rather than in hypothetical conditions. When viewed in this way, the attention shifts to the influence of the environment on the choice. In standard decision science, such an effect is readily explained by a response bias (see Macmillan & Creelman, 2005, Chap. 2), whereby a single decision mechanism produces both responses, but experimental manipulations (e.g., payoff matrices) can change the placement of the response criterion (tending to be more liberal or more conservative). This is a simple, parsimonious conclusion based on decades of decision science.

What emerges from the present discussion is the realization that the field of moral psychology has fixated on the DPMJ and that this has led researchers to make claims about responses, and the processes that produce them, as fact, when they are mere conjecture. Furthermore, this fixation has inhibited any serious discussion of plausible alternative interpretations of the data. This is evident from the fact that, until now, there has been no discussion of the possibility that the effects that Bostyn et al. (2018) reported can be explained simply in terms of well-established concepts in standard decisional science, for example, a change in response bias.

## 6. Psychological Value Theory, a Single-Process Approach

Throughout this critique, we have alluded to a single-process approach that can both accommodate the current data and make novel predictions without relying on the untested assumptions inherent in the DPMJ. To demonstrate that falsifiable, mathematically precise modeling is possible in this domain, we highlight psychological value theory (PVT; e.g., D. J. Cohen & Ahn, 2016; D. J. Cohen et al., 2022, 2023a, 2023b; D. J. Cohen & Quinlan, 2025; Page et al., 2024). PVT is a computational cognitive model of value and choice. While here is not the place to describe the theory in detail (for a detailed presentation, see D. J. Cohen et al., 2022), we provide a brief overview to illustrate how a precise quantified model can account for the data without invoking dual-process binaries.

Psychological value theory (PVT) posits that moral judgments are not the result of a specialized moral cognitive system or a conflict between “emotional” and “rational” processes (as argued by the DPMJ). Instead, PVT argues that moral judgments are general-purpose, value-based preferential choices. The theory proposes that when individuals face a moral dilemma—such as choosing whether to save five people by sacrificing one—they simply attempt to identify and select the option with the highest “Psychological Value.” This value is defined as the perceived importance, worth, or usefulness of an item to the observer. Crucially, PVT treats this value as a perception rather than a static number, meaning it is subject to perceptual variability and is best represented as a distribution of values rather than a single point.

The decision-making process in PVT is computationally modeled using a robust random walk as a sequential sampling model. According to this model, an individual accumulates evidence over time to distinguish between the values of the competing options. The speed and consistency of the decision depend on the distributional overlap of the options’ values. If the values of two options are distinct (low overlap), the decision is fast and consistent; if the values are similar (high overlap), the decision is slow and error-prone. This single stochastic process accurately predicts both the specific choices people make (e.g., saving the group vs. the individual) and the exact time it takes them to decide, accounting for over 90% of the variance in experimental data across various cultures (e.g., D. J. Cohen et al., 2023b) and scenarios (e.g., D. J. Cohen & Ahn, 2016; D. J. Cohen et al., 2022, 2023b; D. J. Cohen & Quinlan, 2025).

PVT explains variations in moral judgments—such as why people are less likely to sacrifice a bystander in the “footbridge” dilemma compared to the standard switch dilemma—through response biases rather than a switch in cognitive systems (e.g., D. J. Cohen & Quinlan, 2025). The theory suggests that factors like “personal force” or cultural norms influence the decision-maker’s starting point or evaluation criterion within the random walk model. These biases reflect a cost–benefit analysis of making an error: for example, the aversion to pushing a person off a bridge is modeled as a bias toward “inaction” (avoiding a “false alarm” of killing the wrong person) rather than considered a deontological rule. Thus, PVT offers a unified consequentialist framework where “utilitarian” and “deontological” responses are simply outcomes of the same value-weighing process under different bias conditions. Such labels do not in any way reflect the operation of different qualitative decisional mechanisms.

## 7. Conclusions

It is without doubt that the contribution Greene and colleagues have made to the field of moral reasoning has been immense, not least because of the amount of work that the theory has generated. More particularly, they have amassed an extensive array of findings that incrementally have been used to support the DPMJ. However, on a careful reading of the literature, we assert that there is very little convincing evidence that provides support for the DPMJ.

The acceptance of the DPMJ has been widespread in both the behavioral (see, e.g., Ahlenius & Tännsjö, 2012; Bostyn et al., 2018; Conway & Gawronski, 2013; Paxton et al., 2012; Suter & Hertwig, 2011; Tinghög et al., 2016; Trémolière & Bonnefon, 2014) and the neuroscience literatures (e.g., Cushman & Greene, 2012; Koenigs et al., 2007; Shenhav & Greene, 2010, 2014). Such work ought to be reassessed in light of the arguments that we have set out here.

There are more recent incarnations of the DPMJ that substitute the rational vs. social/emotional process for other kinds of processes. Because far less research has been directed at these accounts, we cannot provide the same level of analysis as with the DPMJ. For example, Cushman (2013) proposes a dual-process account that consists of a competition between a model-free reinforcement system that computes the value of an action and a model-based reinforcement system that computes the value of a consequence. This and other accounts tend to suffer from the same lack of predictive power as the DPMJ. That is, there are no objective specifications of the activation of each system, their outputs, or their interactions. We recommend that researchers interested in these accounts specify their ideas in quantitative terms so that they produce testable point predictions (e.g., D. J. Cohen et al., 2022).

By way of an alternative approach, Cohen and colleagues (D. J. Cohen & Ahn, 2016; D. J. Cohen et al., 2022) have provided and validated a mathematically precise model of how people solve sacrificial moral dilemmas (namely, psychological value theory, PVT). PVT reveals that people solve moral dilemmas by attempting to save the group with the highest psychological value. Thus, the difficulty of solving the dilemma is gauged to be relative to the similarity in the psychological value of the options rather than a competition between two reasoning processes. Here is not the place to offer a more in-depth account of PVT, but the interested reader is invited to consult the work of Cohen and colleagues directly.

For the readers who remain skeptical that the DPMJ is unfalsifiable, we pose a question: “What is the pattern of data that all DPMJ theorists agree would unequivocally falsify the DPMJ?” Posed another way, “What pattern of data does the DPMJ objectively forbid and why?” If such a pattern cannot be articulated, then the theory cannot be falsified.

## Data Availability

No new data were created or analyzed in this study. Data sharing is not applicable to this article.

## Notes

1 Consistent with the labeling in the extant literature, we will often use the term “utilitarian” or “consequentialist” as shorthand for the cognitive/rational system and “deontology” or “non-consequentialist” as shorthand for the intuitive/affective system. Like many DPMJ researchers, we do not intend to imply a strict interpretation of these terms.

2 Some DPMJ researchers will argue that deontological considerations only come to bear when using someone as a means. This argument, however, inserts an unexplained inconsistency in the DPMJ logic: “Why do some respondents choose to ‘do nothing’ in the switch problem?” There are only two possibilities: (1) an error-prone utilitarian mechanism, or (2) the presence of a third, unidentified process. If either of these premises are accepted, it would be impossible to identify the decision process from the response (see “The DPMJ Assumptions” below). Rather than argue that the deontological process is absent in less emotional dilemmas, most DPMJ researchers argue that it is simply weaker in these dilemmas than in the more emotional dilemmas.

3 In a later discussion, however, Moll and de Oliveira-Souza (2007a) took issue with this nomenclature, claiming that the actual region in question is the medial frontopolar cortex. The region in question ought not to be confused with the more dorsolateral region (BA 46) discussed previously (Greene et al., 2001; Greene & Haidt, 2002).
